# LEEP excision depth is not independently associated with margin positivity or recorded postoperative hrHPV positivity in women with CIN2/3: a retrospective cohort study

**DOI:** 10.3389/fonc.2026.1969282

**Published:** 2026-10-27

**Authors:** Jie Cheng, Chunyan Hu, Genhai Zhu, Fujin Liu, Guifei Li

**Affiliations:** 1 Department of Gynecology, Hainan General Hospital (Hainan Affiliated Hospital of Hainan Medical University), Haikou, China; 2 Department of Pathology, Hainan General Hospital (Hainan Affiliated Hospital of Hainan Medical University), Haikou, China

**Keywords:** cervical intraepithelial neoplasia, excision depth, high-risk human papillomavirus, loop electrosurgical excision procedure, surgical margins

## Abstract

**Objective:**

To examine the relationship between loop electrosurgical excision procedure (LEEP) excision depth and both margin status and postoperative high-risk human papillomavirus (hrHPV) positivity in women treated for cervical intraepithelial neoplasia grades 2–3 (CIN2/3), and to identify clinical factors associated with these outcomes.

**Methods:**

We conducted a retrospective cohort study using deidentified institutional clinical and pathology records from Hainan General Hospital (Hainan Affiliated Hospital of Hainan Medical University). Pathology-recorded LEEP excision depth was evaluated as a continuous variable per 3-mm increment. The primary outcome was positive versus negative excision margin status, and the secondary outcome was recorded postoperative hrHPV positivity. Multivariable logistic regression models were adjusted for age, CIN grade, and preoperative HPV16/18 status. The robustness of the findings was further examined using nonlinear modeling, alternative margin definitions, inverse-probability-of-observation weighting, and interaction analyses. Because postoperative hrHPV testing was non-standardized and outcome data were substantially incomplete, these limitations should be considered when interpreting the findings of the present retrospective investigation.

**Results:**

Among 1, 363 unique patient candidates, 488 had complete data for the core exposure and covariates. The primary margin analysis included 422 women, of whom 41 (9.7%) had positive margins, while the postoperative hrHPV analysis included 275 women, of whom 61 (22.2%) had positive results. There was no clear evidence of an independent association between excision depth and either margin positivity (adjusted odds ratio [OR] per 3 mm, 0.95; 95% CI, 0.72–1.26; P = 0.74) or recorded postoperative hrHPV positivity (adjusted OR, 0.80; 95% CI, 0.57–1.13; *P = 0.*20). In contrast, CIN3 was strongly associated with positive margins (adjusted OR, 3.74; 95% CI, 1.90–7.34; *P < 0.*001), and increasing age was associated with recorded postoperative hrHPV positivity (adjusted OR per 10 years, 1.66; 95% CI, 1.24–2.21; *P < 0.*001). Findings remained consistent across nonlinear, weighted, and alternative-specification analyses.

**Conclusion:**

In this cohort with a relatively narrow range of pathology**-**recorded excision depths, LEEP excision depth provided limited independent statistical information regarding margin positivity and recorded postoperative hrHPV positivity in women with CIN2/3. Histopathologic grade and patient age showed more consistent associations with these outcomes. Within the constraints of this retrospective cohort, these findings do not support reliance on excision depth alone as a marker of treatment adequacy and instead highlight the importance of integrating lesion characteristics, patient-level factors, margin assessment and postoperative HPV surveillance in clinical practice.

## Introduction

Cervical intraepithelial neoplasia grades 2–3 (CIN2/3), the histologic correlate of high-grade squamous intraepithelial lesion (HSIL), represents a clinically important precursor to invasive cervical cancer and remains a major target of cervical cancer prevention programs. Excisional treatment is widely used for high-grade lesions because it allows simultaneous removal of the abnormal transformation zone and histopathologic assessment of the excised specimen. Loop electrosurgical excision procedure (LEEP), also referred to as large loop excision of the transformation zone, is one of the most commonly used techniques for this purpose. Current management increasingly emphasizes risk-based treatment and surveillance, while the procedure itself requires an appropriate balance between adequate lesion removal and preservation of cervical tissue (1–3). This balance is particularly relevant in women of reproductive age, as more extensive excision may improve local disease control but may also increase the risk of subsequent adverse obstetric outcomes, including preterm birth (4).

Among the factors evaluated after excisional treatment, surgical margin status and postoperative high-risk human papillomavirus (hrHPV) testing provide complementary information. Positive margins indicate that dysplastic epithelium may extend to the edge of the excised specimen and are associated with an increased risk of residual or recurrent CIN2 +. Post-treatment hrHPV testing, however, has shown greater sensitivity than margin status for identifying women at risk of treatment failure and has therefore become an important component of post-treatment surveillance (5–7). The interpretation of recorded postoperative HPV positivity is nevertheless influenced by several clinical and virologic factors, including patient age, HPV genotype, testing method, treatment characteristics, and the timing of post-treatment testing (8). These considerations suggest that procedural characteristics should be evaluated together with patient- and disease-related factors rather than considered in isolation.

Excision depth represents one potentially modifiable procedural characteristic of LEEP, yet the achievable depth is constrained by patient age, transformation-zone anatomy, and prior cervical treatment. In clinical practice, a deeper excision may theoretically increase the likelihood of complete removal of endocervical disease, but increasing depth indiscriminately is unlikely to be appropriate because the necessary extent of excision depends on lesion distribution, transformation-zone anatomy, and individual reproductive considerations. Previous studies have reported varying relationships between excision depth and treatment outcomes. Several cohorts have suggested that greater excision depth or transformation-zone-specific excision is associated with a higher probability of negative margins (9–11). In contrast, other studies have shown satisfactory disease control and HPV-related outcomes after relatively conservative excision, including excision depths of 10 mm or less (12). More recent evidence has also suggested that margin clearance may provide more clinically relevant information for test-of-cure outcomes than excision depth alone (13).

These findings indicate that the clinical relevance of excision depth may depend on the population studied and on the accompanying pathological and virologic characteristics. In particular, it remains uncertain whether pathology-recorded excision depth contributes independent information regarding margin involvement and recorded postoperative hrHPV positivity after readily available clinical factors are taken into account. Therefore, we evaluated the associations of LEEP excision depth with margin positivity and recorded postoperative hrHPV positivity in a retrospective cohort of women treated for CIN2/3. We further examined whether these associations were influenced by clinically relevant patient and lesion characteristics. We hypothesized that greater excision depth would be associated with lower odds of positive margins and recorded postoperative hrHPV positivity.

## Methods

### Study design and participants

This retrospective cohort study included women who underwent loop electrosurgical excision procedure (LEEP) for newly-diagnosed cervical intraepithelial neoplasia grades 2–3 (CIN2/3) at Hainan General Hospital (Hainan Affiliated Hospital of Hainan Medical University) between April 2018 and November 2025. Patients with prior cervical excisional treatment for recurrent CIN were excluded. This single-center study was reported in accordance with the Strengthening the Reporting of Observational Studies in Epidemiology (STROBE) statement (14). Ethical approval for the study protocol was granted by the Ethics Committee. Clinical, virologic, and pathological data were obtained from deidentified institutional records. Records referring to the same patient were consolidated using a unique deidentified patient identifier. Eligible patients were required to have a definitive histopathologic diagnosis of CIN2 or CIN3, an available pathology-recorded LEEP excision depth, age information, and a documented preoperative HPV result. Patients with unresolved or conflicting histopathologic grades, unavailable or uninterpretable excision-depth measurements, or missing core covariates were excluded from the core analytical cohort.

Because reliable dates of residual or recurrent disease and corresponding censoring information were not consistently available, the present analysis focused on margin status and recorded postoperative hrHPV positivity as the principal study outcomes. Outcome-specific cohorts were subsequently constructed according to the availability of margin status and postoperative high-risk human papillomavirus (hrHPV) results. Patients with an evaluable margin status were included in the primary margin analysis, whereas those with an available postoperative hrHPV result were included in the secondary analysis. The two analytical cohorts were therefore allowed to overlap. The study flow is shown in **Figure 1**.

**Figure 1:**
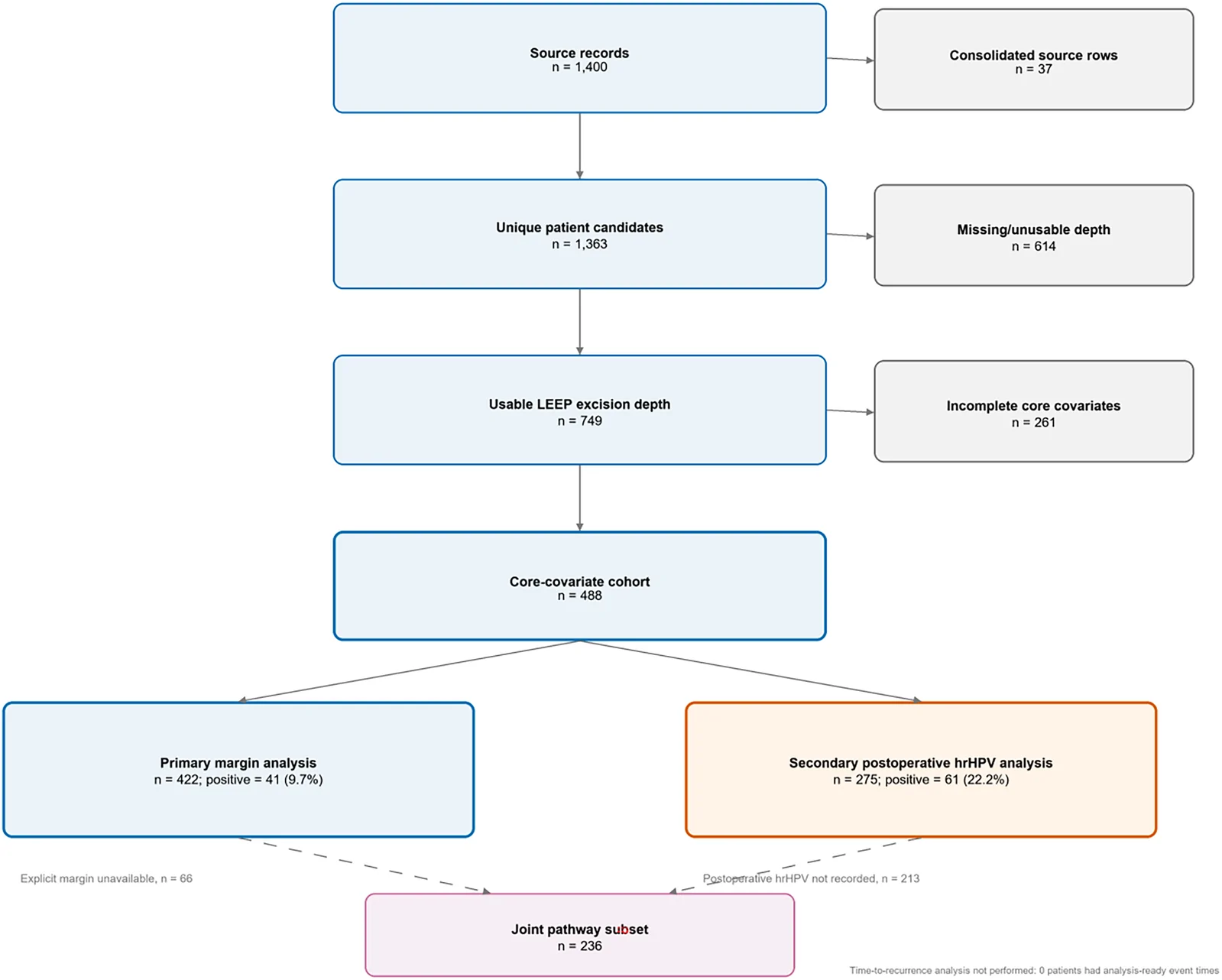
Study cohort and outcome-specific analytical samples. The 1, 400 source records were consolidated into 1, 363 unique patient candidates. After excluding patients without usable depth or complete core covariates, 488 formed the core-covariate cohort. Primary margin and secondary postoperative hrHPV analyses were conducted in overlapping outcome-specific samples. The joint pathway subset contained patients with both recorded postoperative hrHPV results and positive-or-unassessable margin status available. No time-to-recurrence analysis was performed because no patient had an analysis-ready event time.

### LEEP excision depth

The primary exposure was the LEEP excision depth recorded in the pathology-derived dataset. Excision depth was expressed in millimeters and analyzed primarily as a continuous variable. To improve clinical interpretability, effect estimates were reported per 3-mm increase in excision depth.

For fragmented or multiple submitted tissue specimens, pathologists reconstructed tissue and recorded the maximum measured depth across all fragments. Patients receiving separate top-hat or supplementary endocervical excision were excluded, given that such additional resection would alter recorded specimen depth and would not reflect primary LEEP excision performance.

For analyses examining the robustness of the depth–margin association, alternative upper limits of 20, 30, and 50 mm were applied to reduce the potential influence of unusually large recorded values. These thresholds were selected based on previously reported depth distributions from published LEEP cohorts to evaluate whether extreme recorded depth values could alter our main conclusions. The full observed range of excision depth was 2–35 mm; because no value exceeded 50 mm, the 50**-**mm upper bound was never triggered. Observations exceeding each upper bound were capped at the corresponding threshold. Information regarding loop size, operator**-**related variation, and additional resection was not consistently captured for this cohort.

### Study outcomes

The primary outcome was positive excision margin status. Close margin was defined as dysplastic epithelium within 1 mm of the specimen resection edge according to local pathology reporting practice. Margins explicitly documented as positive or involved were classified as positive, whereas margins explicitly documented as negative were classified as negative. Margins reported as close, unassessable, unknown, or incomplete were not included in the primary margin definition. Standardized separate reporting for endocervical, ectocervical, and deep margins was inconsistently available across pathology records; hence stratified analyses by individual margin anatomical location could not be performed.

Because margin classification may vary according to pathological reporting and clinical interpretation, two additional definitions were examined in sensitivity analyses. In the first alternative definition, unassessable margins were grouped with positive margins. In the broader definition, both unassessable and close margins were classified as positive.

The secondary outcome was recorded postoperative hrHPV positivity. A postoperative HPV result was classified as positive when HPV16/18, another high-risk HPV genotype, or an unspecified high-risk HPV-positive result was recorded. Patients with a documented negative postoperative hrHPV result were classified as negative.

Many patients were lost to follow-up and did not complete preoperative and postoperative hrHPV testing at our institution for various reasons. Archived clinical records do not consistently store full genotype-specific outputs; thus pairwise pre-versus postoperative HPV genotype concordance could not be assessed. For participants with multiple available postoperative hrHPV records, only the first documented postoperative hrHPV test result was used for analysis. Exact time intervals between LEEP and postoperative hrHPV testing were inconsistently captured in hospital records; for this reason, the secondary endpoint was defined as recorded postoperative hrHPV positivity and not prespecified-time-point HPV persistence. Postoperative hrHPV genotyping was performed using the HybriMax nucleic acid hybridization system (Hybribio, Chaozhou, China). The HPV genotyping assay was the 37-type human papillomavirus nucleic acid amplification and genotyping kit (PCR and membrane hybridization method, Hybribio). Preoperative HPV testing was performed using this identical assay platform. Some historical laboratory records only provided pooled high-risk-HPV positive or negative results and lacked separate outputs for individual genotypes. Therefore, recorded postoperative hrHPV positivity serves only as an exploratory marker of viral detection, and cannot be interpreted as definitively verified persistent HPV infection; incident new HPV infections cannot be excluded.

### Covariates

Core covariates were selected *a priori* according to clinical relevance and availability in the source dataset. These included age, histopathologic grade, and preoperative HPV16/18 status. Age was analyzed continuously and scaled per 10-year increase. Histopathologic grade was categorized as CIN3 versus CIN2. Preoperative HPV status was categorized as HPV16/18 positive versus other high-risk HPV types or negative HPV results. Preoperative HPV testing was performed with the same assay platform used for postoperative testing, namely the HybriMax nucleic acid hybridization system (Hybribio, Chaozhou, China) and the 37-type human papillomavirus nucleic acid amplification and genotyping kit (PCR and membrane hybridization method, Hybribio).

Additional variables considered in extended models included high-grade cytology and gland involvement. High-grade cytology was defined as atypical squamous cells, cannot exclude high-grade squamous intraepithelial lesion (ASC-H), high-grade squamous intraepithelial lesion (HSIL), or a more severe cytologic abnormality.

For analyses of recorded postoperative hrHPV positivity, margin status was additionally incorporated to examine whether the association between excision depth and recorded postoperative hrHPV remained after accounting for pathological margin findings.

Prespecified potential effect modifiers included histopathologic grade, preoperative HPV16/18 status, gland involvement, high-grade cytology, and age ≥40 years.

### Statistical analysis

Continuous variables were summarized as medians with interquartile ranges (IQRs), and categorical variables were presented as numbers and percentages. Baseline characteristics were compared according to margin status, according to inclusion in versus exclusion from the core cohort (**Supplementary Table S2**), and according to the availability of postoperative hrHPV data (**Supplementary Table S3**) using the Mann–Whitney U test for continuous variables and the chi-square test or Fisher exact test for categorical variables, as appropriate. These comparisons were descriptive and were not used for covariate selection. Covariates were not treated as secondary study outcomes.

Associations between LEEP excision depth and each study outcome were evaluated using logistic regression. Odds ratios (ORs) and 95% confidence intervals (CIs) were reported.

For the primary margin outcome, an unadjusted model was first fitted with excision depth as the exposure. The core multivariable model included excision depth, age, histopathologic grade, and preoperative HPV16/18 status. An extended model additionally incorporated high-grade cytology and gland involvement.

The same modeling strategy was used for recorded postoperative hrHPV positivity. In addition, a margin-adjusted model was constructed by including positive or unassessable margin status together with excision depth, age, histopathologic grade, and preoperative HPV16/18 status. An extended margin-adjusted model further included high-grade cytology and gland involvement.

All covariates were entered simultaneously according to the prespecified analytical framework, without stepwise selection. Model stability was assessed by examining convergence and the number of outcome events relative to the number of model parameters. Multicollinearity was evaluated using variance inflation factors (VIFs), with VIF <5 considered acceptable. Given the retrospective design, all eligible patients meeting the study criteria during the predefined study period were included in the analysis.

#### Missing data and outcome-observation weighting

Regression analyses were performed using model-specific complete cases. This approach allowed patients to remain eligible for models that did not require variables missing from other analyses. Study outcomes were not imputed. Because margin status and postoperative hrHPV results were not available for all patients in the core-covariate cohort, inverse-probability-of-observation weighting (IPOW) was applied as a sensitivity analysis to assess potential bias introduced by differential outcome ascertainment. Baseline characteristics comparing included versus excluded participants are reported in **Supplementary Table S2**.

For each outcome, the probability of having the outcome recorded was estimated using excision depth, age, histopathologic grade, and preoperative HPV16/18 status. Stabilized inverse-probability weights were then calculated and truncated at the 1st and 99th percentiles. The core logistic regression models were subsequently refitted using these weights with robust standard errors. By re-weighting the analytical sample according to the estimated probability of outcome observation, IPOW explicitly targeted the differential, covariate-dependent ascertainment of both outcomes. The weighted depth estimates remained virtually identical to the complete-case estimates (0.96 vs 0.95 per 3 mm for margin positivity; 0.78 vs 0.80 per 3 mm for recorded postoperative hrHPV positivity), indicating that differential follow-up related to measured covariates did not materially distort the primary estimates.

#### Assessment of nonlinear associations

Potential nonlinear associations between LEEP excision depth and the two study outcomes were examined using natural cubic spline models with 3 degrees of freedom. The fit of the spline model was compared with that of the corresponding linear model using a likelihood-ratio test. To limit the influence of sparsely represented extreme values, the graphical presentation of the spline analyses was restricted to the 2nd through 98th percentiles of the observed excision-depth distribution. Observed excision depth in the core-covariate cohort ranged from **2** mm to **35** mm.

#### Sensitivity and specification analyses

To assess the robustness of the association between excision depth and margin positivity, a specification analysis was performed across 27 analytically reasonable model combinations. These combinations encompassed three definitions of margin positivity, three upper-bound thresholds for excision depth (20, 30, and 50 mm), and three covariate-adjustment levels: unadjusted, core-adjusted, and extended-adjusted. The Benjamini–Hochberg procedure was applied to control the false-discovery rate across the 27 model specifications. A sensitivity analysis examining the composition of the preoperative HPV reference group showed that excluding the 7 preoperatively HPV-negative patients (1.7% of the cohort) from the pooled non-16/18 reference group left the depth-related effect estimate unchanged (adjusted OR, 0.95; 95% CI, 0.72–1.26; P = 0.74) (**Supplementary Table S1**).

#### Effect-modification analyses

Potential effect modification was evaluated by introducing interaction terms between excision depth and each prespecified clinical factor. These factors included CIN grade, preoperative HPV16/18 status, gland involvement, high-grade cytology, and age group (<40 vs ≥40 years).

Interaction analyses were performed separately for margin positivity and recorded postoperative hrHPV positivity, resulting in 10 interaction models. False-discovery-rate correction using the Benjamini–Hochberg method was applied to the interaction tests.

#### Exploratory pathway analysis

An exploratory pathway analysis was performed among patients with both margin status and recorded postoperative hrHPV results available to examine the extent to which the association between excision depth and recorded postoperative hrHPV might be related to margin status.

A clinically interpretable contrast of 8-mm versus 5-mm excision depth was selected. Separate logistic regression models were fitted for margin status and recorded postoperative hrHPV positivity, with adjustment for age, histopathologic grade, and preoperative HPV16/18 status.

Standardization was used to estimate the absolute difference in the predicted probability of recorded postoperative hrHPV positivity between the two depth levels. The analysis generated estimates corresponding to the total association, the association after accounting for margin status, and the margin-associated indirect component. Confidence intervals were obtained using 1, 000 nonparametric bootstrap samples.

Because the analysis was based on observational data and postoperative HPV testing intervals were not standardized, these estimates were interpreted as exploratory pathway associations rather than causal mediation effects.

#### Statistical software

All statistical analyses and data visualization were performed using R software (version 4.4.2; R Foundation for Statistical Computing, Vienna, Austria). All statistical tests were two-sided. A *P value < 0.05* was considered statistically significant, except for analyses in which false-discovery-rate-adjusted *q* values were reported.

## Results

### Study population and baseline characteristics

A total of 1, 400 source records were identified and consolidated into 1, 363 unique patients. Among these, 749 patients had an available LEEP excision-depth measurement, and 488 had complete data for the core exposure and covariates. The primary margin analysis included 422 patients with evaluable margin status (derived from a broader pool of 643 patients with available depth and explicit margin information; n = 422 required complete core covariates), of whom 41 (9.7%) had positive margins. The unadjusted analysis for recorded postoperative hrHPV positivity included 387 patients; the core multivariable postoperative hrHPV analysis included 275 patients with complete core covariates and an available recorded postoperative hrHPV result, including 61 (22.2%) with a positive result. A total of 236 patients had both margin and recorded postoperative hrHPV information available and were included in the exploratory pathway analysis (**Figure 1**).

In the primary margin-analysis cohort, the median age was 39 years (IQR, 34–47 years), and the median LEEP excision depth was 6 mm (IQR, 5–8 mm**;** full observed range, 2–35 mm). Overall, 111 patients (26.3%) had CIN3 and 311 (73.7%) had CIN2.

Patients with positive margins were older than those with negative margins (median, 45 vs 38 years; P = 0.01), and CIN3 was more frequently observed among patients with positive margins (56.1% vs 23.1%; P < 0.001). Gland involvement was also more common in the positive-margin group (75.6% vs 59.1%; P = 0.04). In contrast, the median excision depth was identical in the two groups (6 mm [IQR, 5–8 mm] in both groups; P = 0.77). The distributions of other baseline characteristics are summarized in **Table 1**, and the distribution of excision depth, availability of key variables, and observed outcome proportions are shown in **Figure 2**.

**Table 1:** Characteristics of the primary margin-analysis cohort.

Characteristic	Available, no.	Overall (N = 422)	Negative margin (n = 381)	Positive margin (n = 41)	*P* value
Age, y	422	39.0 (34.0–47.0)	38.0 (33.0–46.0)	45.0 (37.0–52.0)	0.01
LEEP excision depth, mm	422	6.0 (5.0–8.0)	6.0 (5.0–8.0)	6.0 (5.0–8.0)	0.77
CIN3 vs CIN2, No. (%)	422	111 (26.3)	88 (23.1)	23 (56.1)	<0.001
Preoperative HPV16/18, No. (%)	422	181 (42.9)	160 (42.0)	21 (51.2)	0.26
Gland involvement, No. (%)	420	255 (60.7)	224 (59.1)	31 (75.6)	0.04

Data are median (IQR) unless otherwise indicated. Percentages use the available denominator within each margin group. *P* values were calculated with the Mann–Whitney U test for continuous variables and the chi-square or Fisher exact test for categorical variables, as appropriate. CIN, cervical intraepithelial neoplasia; HPV, human papillomavirus; IQR, interquartile range; LEEP, loop electrosurgical excision procedure. High-grade cytology was evaluated in the extended multivariable model (**Table 2**).

**Figure 2:**
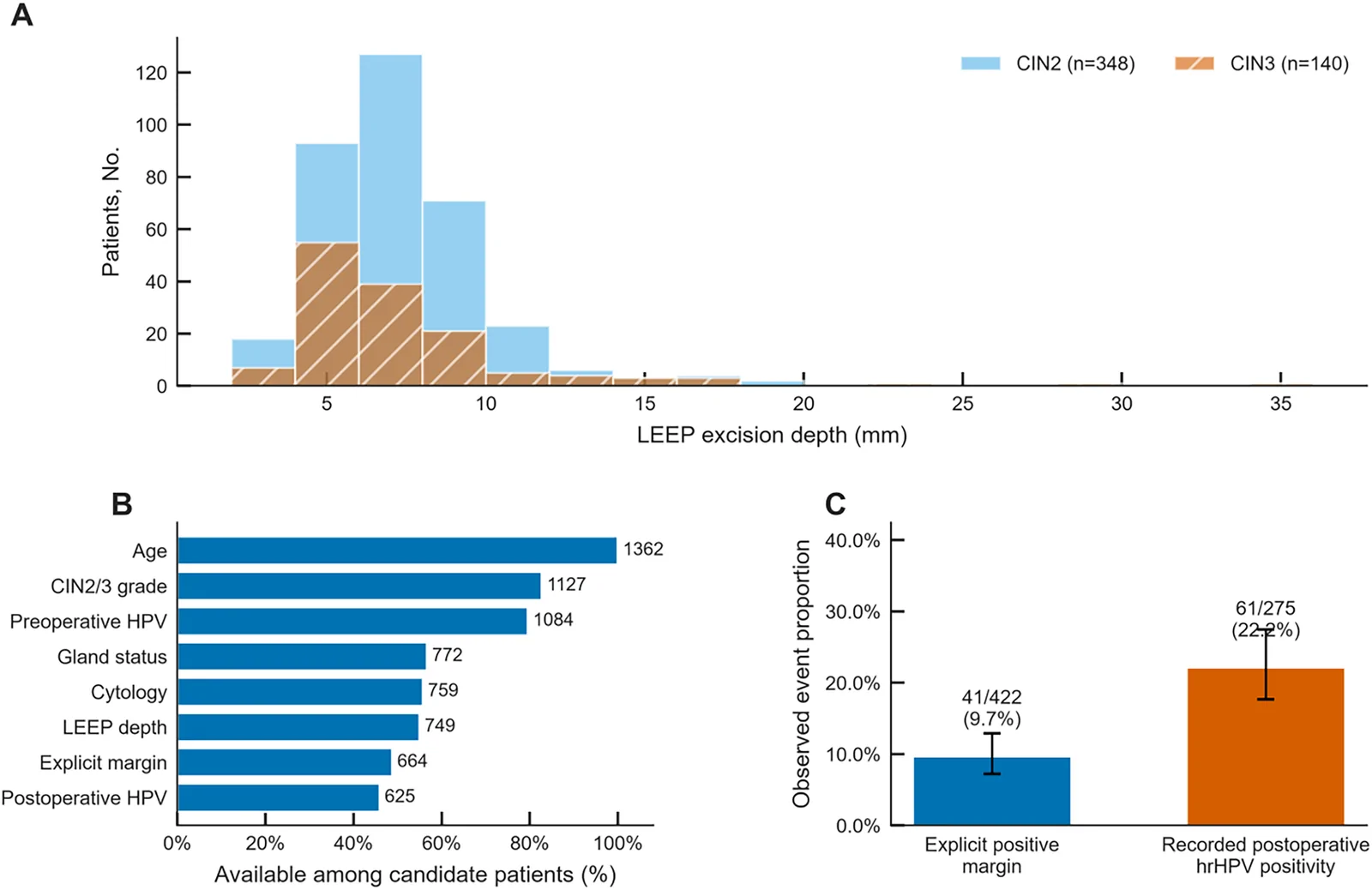
Exposure distribution, data availability, and observed outcome proportions. **(A)** Distribution of pathology-recorded LEEP excision depth in the 488-patient core-covariate cohort, stratified by CIN2 or CIN3. **(B)** Availability of key variables among all 1, 363 unique patient candidates; numbers at the end of bars are observed counts. **(C)** Proportions of explicit positive margins and recorded postoperative hrHPV-positive results in the core adjusted analytical samples. Error bars are Wilson 95% CIs. CIN, cervical intraepithelial neoplasia; hrHPV, high-risk human papillomavirus; LEEP, loop electrosurgical excision procedure.

Within the core-covariate cohort, baseline characteristics were directly compared between the 275 patients with recorded postoperative hrHPV data and the 213 patients without such data (**Supplementary Table S3**). The two groups were similar with respect to age (median, 39.0 [IQR, 34.0–47.0] vs 39.0 [IQR, 33.0–50.0] years; P = 0.948), histopathologic grade (CIN3, 29.1% vs 28.2%; P = 0.823), preoperative HPV16/18 positivity (40.4% vs 45.5%; P = 0.251), gland involvement (60.1% vs 65.4% among patients with available data; P = 0.237), and high-grade cytology (31.0% vs 34.8% among patients with available data; P = 0.463). The only measured difference was LEEP excision depth, which was modestly greater among patients with recorded postoperative hrHPV data (median, 7.0 [IQR, 5.0–8.0] vs 6.0 [IQR, 5.0–8.0] mm; P = 0.001), a pattern consistent with more intensive documentation or follow-up among patients undergoing more extensive procedures. This measured imbalance was explicitly addressed in the inverse-probability-of-observation-weighted sensitivity analyses; unmeasured determinants of follow-up, however, cannot be ruled out (see Limitations).

### Association between excision depth and margin positivity

In the unadjusted analysis of 643 patients (72 margin-positive events) with available depth and explicit margin information, the estimated association between excision depth and margin positivity was not statistically significant (OR per 3-mm increase, 0.98; 95% CI, 0.78–1.23; *P = 0.*85).

After adjustment for age, histopathologic grade, and preoperative HPV16/18 status (n = 422; 41 events), the effect estimate remained similar (adjusted OR per 3-mm increase, 0.95; 95% CI, 0.72–1.26; P = 0.74). Further adjustment for high-grade cytology and gland involvement (n = 283; 33 events) produced a comparable estimate (adjusted OR, 0.93; 95% CI, 0.67–1.30; P = 0.68) (**Table 2**; **Figure 3A**).

**Table 2:** Logistic regression models for margin and recorded postoperative hrHPV positivity.

Outcome and model	N (events)	Covariate	OR (95% CI)	*P* value
Margin, unadjusted	643 (72)	Depth, per 3 mm	0.98 (0.78–1.23)	0.85
Margin, core adjusted	422 (41)	Depth, per 3 mm	0.95 (0.72–1.26)	0.74
Margin, core adjusted	422 (41)	Age, per 10 y	1.35 (1.00–1.82)	0.047
Margin, core adjusted	422 (41)	CIN3 vs CIN2	3.74 (1.90–7.34)	<0.001
Margin, core adjusted	422 (41)	Preoperative HPV16/18	1.41 (0.71–2.77)	0.32
Margin, extended adjusted	283 (33)	Depth, per 3 mm	0.93 (0.67–1.30)	0.68
Margin, extended adjusted	283 (33)	Age, per 10 y	1.13 (0.81–1.60)	0.47
Margin, extended adjusted	283 (33)	CIN3 vs CIN2	2.73 (1.17–6.34)	0.02
Margin, extended adjusted	283 (33)	Preoperative HPV16/18	1.17 (0.54–2.54)	0.69
Margin, extended adjusted	283 (33)	High-grade cytology	1.29 (0.57–2.91)	0.54
Margin, extended adjusted	283 (33)	Gland involvement	0.96 (0.39–2.39)	0.94
Recorded postoperative hrHPV, unadjusted	387 (83)	Depth, per 3 mm	0.75 (0.56–1.01)	0.06
Recorded postoperative hrHPV, core adjusted	275 (61)	Depth, per 3 mm	0.80 (0.57–1.13)	0.20
Recorded postoperative hrHPV, core adjusted	275 (61)	Age, per 10 y	1.66 (1.24–2.21)	<0.001
Recorded postoperative hrHPV, core adjusted	275 (61)	CIN3 vs CIN2	0.75 (0.38–1.46)	0.39
Recorded postoperative hrHPV, core adjusted	275 (61)	Preoperative HPV16/18	1.21 (0.66–2.22)	0.54
Recorded postoperative hrHPV, margin adjusted	236 (55)	Depth, per 3 mm	0.72 (0.49–1.06)	0.09
Recorded postoperative hrHPV, margin adjusted	236 (55)	Positive/unassessable margin	1.45 (0.56–3.75)	0.45
Recorded postoperative hrHPV, margin adjusted	236 (55)	Age, per 10 y	1.84 (1.33–2.54)	<0.001
Recorded postoperative hrHPV, margin adjusted	236 (55)	CIN3 vs CIN2	0.81 (0.38–1.71)	0.58
Recorded postoperative hrHPV, margin adjusted	236 (55)	Preoperative HPV16/18	1.20 (0.62–2.29)	0.59
Recorded postoperative hrHPV, extended margin adjusted	165 (40)	Depth, per 3 mm	0.93 (0.64–1.35)	0.71
Recorded postoperative hrHPV, extended margin adjusted	165 (40)	Positive/unassessable margin	1.35 (0.45–4.05)	0.60
Recorded postoperative hrHPV, extended margin adjusted	165 (40)	Age, per 10 y	1.92 (1.30–2.84)	0.001
Recorded postoperative hrHPV, extended margin adjusted	165 (40)	CIN3 vs CIN2	0.60 (0.21–1.69)	0.34
Recorded postoperative hrHPV, extended margin adjusted	165 (40)	Preoperative HPV16/18	1.52 (0.69–3.35)	0.30
Recorded postoperative hrHPV, extended margin adjusted	165 (40)	High-grade cytology	1.67 (0.69–4.03)	0.26
Recorded postoperative hrHPV, extended margin adjusted	165 (40)	Gland involvement	0.55 (0.24–1.26)	0.16

ORs are from logistic regression with model-based 95% CIs. Core models included depth, age, grade, and preoperative HPV16/18. Extended models additionally included high-grade cytology and gland involvement. Margin-adjusted recorded postoperative hrHPV models included positive or unassessable margins. CIN, cervical intraepithelial neoplasia; hrHPV, high-risk human papillomavirus; OR, odds ratio.

**Figure 3:**
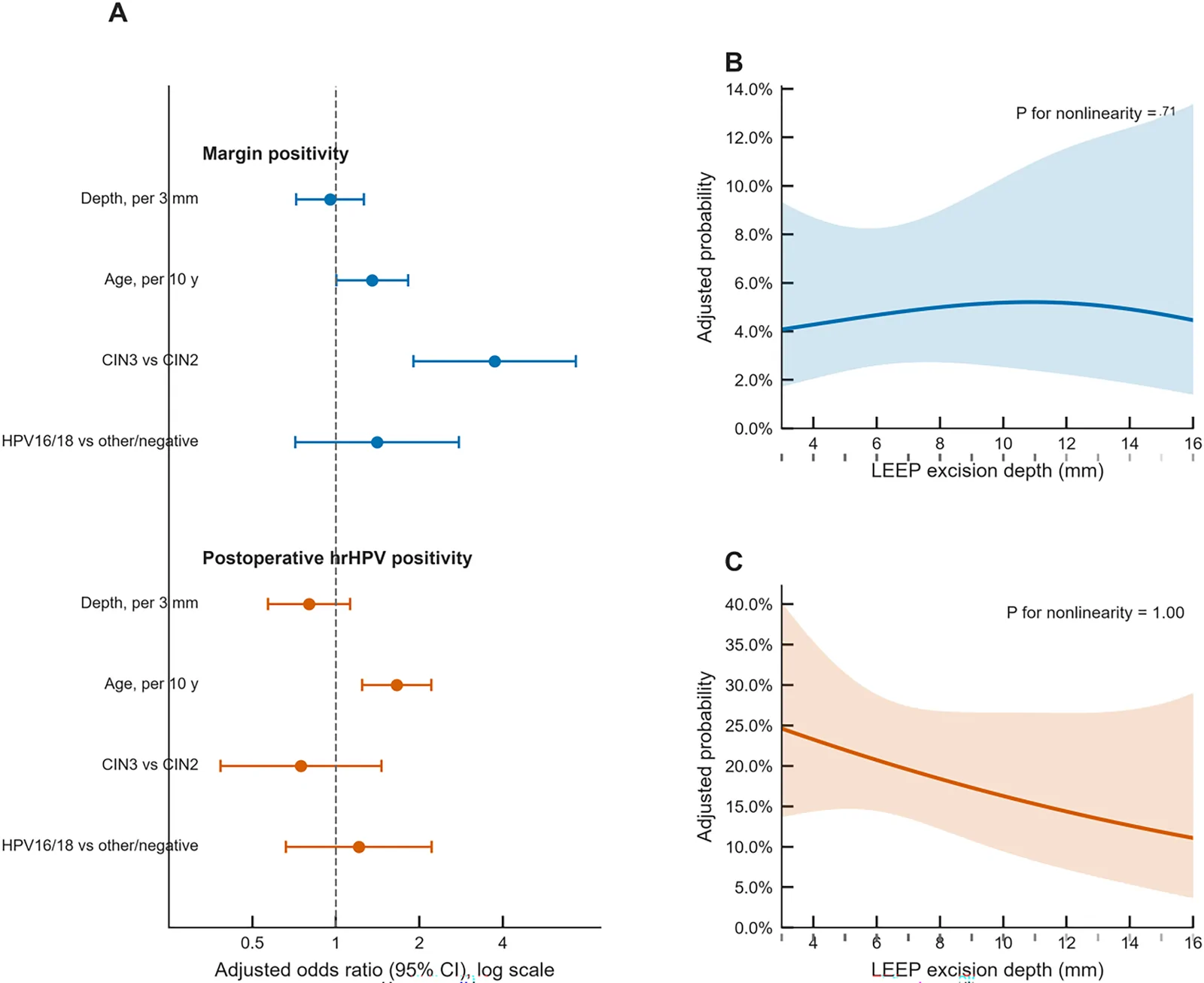
Adjusted associations of excision depth and clinical covariates with margin and postoperative hrHPV positivity. **(A)** Core multivariable logistic regression estimates for explicit margin positivity (n = 422; 41 events) and postoperative hrHPV positivity (n = 275; 61 events). Points are adjusted ORs and horizontal lines are 95% CIs; the dashed line denotes OR = 1. **(B, C)** Natural cubic spline estimates for margin positivity and postoperative hrHPV positivity, respectively, for a reference patient with median age, CIN2, and no preoperative HPV16/18. Curves are restricted to the 2nd–98th percentile of observed depth; shading denotes 95% CIs and rugs show observed depths within the plotted range. CIN, cervical intraepithelial neoplasia; hrHPV, high-risk human papillomavirus; OR, odds ratio.

Among the clinical covariates included in the core model, CIN3 showed the strongest association with margin positivity. Compared with CIN2, CIN3 was associated with approximately 3.7-fold higher odds of a positive margin (adjusted OR, 3.74; 95% CI, 1.90–7.34; *P < 0.*001). Increasing age was also associated with higher odds of margin positivity, although the magnitude of this association was smaller (adjusted OR per 10-year increase, 1.35; 95% CI, 1.00–1.82; *P = 0.*047). Preoperative HPV16/18 status was not independently associated with margin positivity in the core model (adjusted OR, 1.41; 95% CI, 0.71–2.77; *P = 0.*32).

### Association between excision depth and postoperative hrHPV positivity

Among 387 patients (83 events) included in the unadjusted recorded postoperative hrHPV analysis, the unadjusted OR for a 3-mm increase in excision depth was 0.75; 95% CI, 0.56–1.01; P = 0.06.

In the core multivariable model including 275 patients and 61 recorded postoperative hrHPV-positive events, the adjusted OR for a 3-mm increase in excision depth was 0.80 (95% CI, 0.57–1.13; P = 0.20). Age demonstrated a more consistent association with recorded postoperative hrHPV status: each 10-year increase in age was associated with higher odds of recorded postoperative hrHPV positivity (adjusted OR, 1.66; 95% CI, 1.24–2.21; P < 0.001). Neither histopathologic grade nor preoperative HPV16/18 status was independently associated with recorded postoperative hrHPV positivity in this model (**Table 2**; **Figure 3A**).

Among the 236 patients (55 events) with both margin and recorded postoperative hrHPV data available, additional adjustment for positive or unassessable margins yielded a depth estimate of 0.72 per 3-mm increase (95% CI, 0.49–1.06; P = 0.09). Margin status itself showed an adjusted OR of 1.45 for recorded postoperative hrHPV positivity (95% CI, 0.56–3.75; P = 0.45). In this model, age remained consistently associated with recorded postoperative hrHPV positivity (adjusted OR per 10-year increase, 1.84; 95% CI, 1.33–2.54; P < 0.001).

The extended margin-adjusted model, which additionally included high-grade cytology and gland involvement (n = 165; 40 events), produced a similar overall pattern, with an adjusted OR of 0.93 for excision depth (95% CI, 0.64–1.35; *P = 0.*71) and 1.92 for age per 10-year increase (95% CI, 1.30–2.84; *P = 0.*001) (**Table 2**).

### Nonlinear and robustness analyses

Natural cubic spline analyses showed no meaningful departure from a linear relationship between excision depth and either study outcome. The likelihood-ratio test for nonlinearity was P = 0.71 for margin positivity and P = 1.00 for recorded postoperative hrHPV positivity (**Figures 3B, C**).

Results from the inverse-probability-of-observation-weighted analyses were closely aligned with those from the complete-case models. For margin positivity, the weighted OR per 3-mm increase in excision depth was 0.96 (95% CI, 0.76–1.21; *P = 0.*72), compared with 0.95 (95% CI, 0.72–1.26; *P = 0.*74) in the complete-case analysis. For recorded postoperative hrHPV positivity, the corresponding estimates were 0.78 (95% CI, 0.54–1.14; P = 0.20) and 0.80 (95% CI, 0.57–1.13; P = 0.20), respectively (**Table 3**; **Figure 4B**). This indicates that differential follow-up related to measured covariates did not materially distort the primary estimates.

**Table 3:** Robustness, nonlinearity, and exploratory pathway analyses.

Analysis	Outcome or contrast	N	Estimate (95% CI)	*P* or *q* value	Interpretation
Natural cubic spline	Margin positivity	422	LR χ² = 0.69	0.71	*P* for nonlinearity
Natural cubic spline	Recorded postoperative hrHPV positivity	275	LR χ² < 0.01	1.00	*P* for nonlinearity
Complete case	Margin positivity	422	OR 0.95 (0.72–1.26)	0.74	Per 3-mm greater depth
IPOW	Margin positivity	422	OR 0.96 (0.76–1.21)	0.72	Per 3-mm greater depth
Complete case	Recorded postoperative hrHPV positivity	275	OR 0.80 (0.57–1.13)	0.20	Per 3-mm greater depth
IPOW	Recorded postoperative hrHPV positivity	275	OR 0.78 (0.54–1.14)	0.20	Per 3-mm greater depth
Specification curve	27 margin specifications	280–700	Median OR 0.95; range, 0.82–1.09	0/27 with *q < 0.05*	BH correction across models
Effect modification	Smallest *q*: margin × CIN3	422	Interaction OR 1.62 (0.77–3.39)	0.63	BH-corrected *q* value
Associational pathway	Total association	236	−5.80 (−15.31 to −0.43) percentage points	NA	8-mm vs 5-mm contrast
Associational pathway	Direct association	236	−5.71 (−15.12 to −0.53) percentage points	NA	8-mm vs 5-mm contrast
Associational pathway	Indirect association through margin	236	−0.08 (−0.67 to 0.32) percentage points	NA	8-mm vs 5-mm contrast
Collinearity	Core models	NA	Maximum VIF, 1.06	NA	VIF <5 prespecified as acceptable

The specification curve combined three margin definitions, three depth caps, and three adjustment sets. IPOW used stabilized weights truncated at the 1st and 99th percentiles and heteroscedasticity-consistent standard errors. Pathway estimates are standardized absolute differences with 1, 000-sample percentile bootstrap CIs and are associational, not causal. BH, Benjamini–Hochberg; IPOW, inverse probability of observation weighting; LR, likelihood ratio; NA, not applicable; OR, odds ratio; VIF, variance inflation factor.

**Figure 4:**
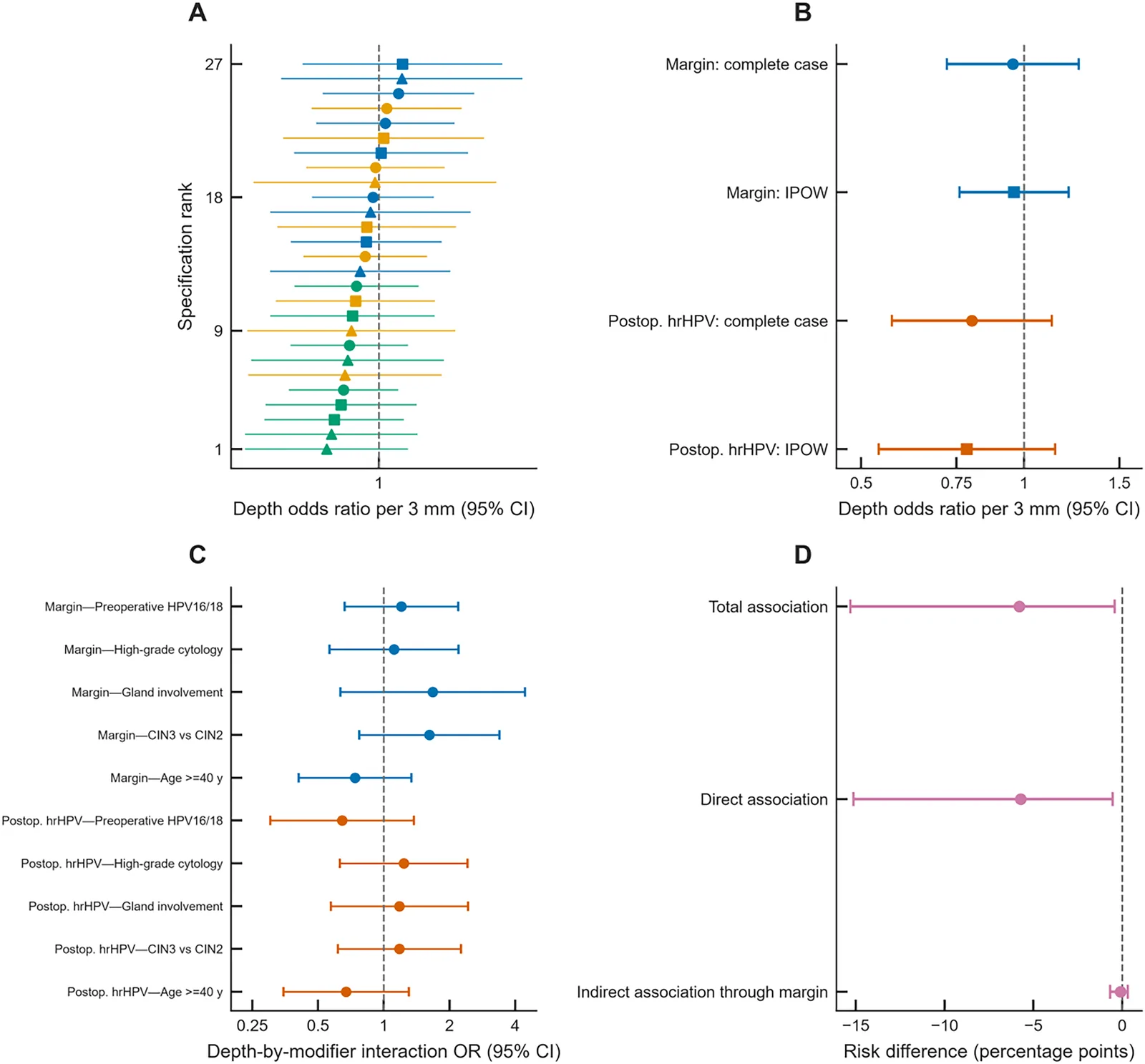
Robustness, effect-modification, and exploratory pathway analyses. **(A)** Depth estimates across 27 defensible margin specifications. Blue, orange, and green denote explicit-positive, positive-or-unassessable, and broad margin definitions; circles, squares, and triangles denote unadjusted, core, and extended adjustment sets. **(B)** Complete-case and inverse-probability-of-observation-weighted depth estimates for both outcomes. **(C)** Depth-by-modifier interaction estimates; no interaction remained significant after Benjamini–Hochberg correction. **(D)** Standardized absolute differences in postoperative hrHPV probability for 8-mm versus 5-mm depth in the joint subset. The total and direct contrasts were negative, whereas the margin-associated indirect contrast was near zero. The pathway analysis is associational and should not be interpreted as causal mediation. hrHPV, high-risk human papillomavirus; IPOW, inverse probability of observation weighting; OR, odds ratio.

The specification analysis further evaluated the depth–margin relationship across 27 combinations of margin definitions, excision-depth limits, and adjustment strategies. The median OR across these models was 0.95, with individual estimates ranging from 0.82 to 1.09. None of the 27 specifications met the false-discovery-rate-adjusted threshold for statistical significance (**Figure 4A**).

Effect-modification analyses also showed broadly consistent depth estimates across the prespecified clinical subgroups. After Benjamini–Hochberg correction, none of the 10 interaction tests reached statistical significance; the smallest adjusted *q* value was 0.63 (**Table 3**; **Figure 4C**).

### Exploratory pathway analysis

The exploratory pathway analysis included 236 patients with both margin and recorded postoperative hrHPV information available. When an excision depth of 8 mm was compared with 5 mm, the standardized total difference in the predicted probability of recorded postoperative hrHPV positivity was −5.80 percentage points (95% bootstrap CI, −15.31 to −0.43).

After accounting for margin status, the corresponding direct difference was −5.71 percentage points (95% CI, −15.12 to −0.53). The margin-associated indirect component was small, at −0.08 percentage points (95% CI, −0.67 to 0.32) (**Table 3**; **Figure 4D**).

All logistic regression models converged successfully. The number of events per nonintercept parameter was 10.25 for the core margin model and 15.25 for the core recorded postoperative hrHPV model. The maximum variance inflation factor was 1.06, indicating minimal multicollinearity among the covariates included in the core models.

## Discussion

In this retrospective cohort of women treated with LEEP for CIN2/3, there was no clear statistical evidence of an independent association between excision depth and either margin positivity or recorded postoperative hrHPV positivity after adjustment for clinically relevant factors. While multiple sensitivity analyses yielded consistent point estimates, these cannot fully overcome limitations inherent to retrospective observational data. In contrast, histopathologic grade was more strongly associated with margin status, whereas increasing age was consistently associated with recorded postoperative hrHPV positivity. Taken together, these findings suggest that excision depth represents only one component of treatment adequacy and postoperative risk assessment and should be interpreted alongside lesion characteristics and patient-level factors.

Prior published studies have reported conflicting findings regarding the clinical impact of LEEP excision depth. Some studies demonstrated that deeper excision improved margin clearance, whereas other cohorts achieved acceptable oncological outcomes with conservative excision depth. Most previous investigations dichotomized excision depth using arbitrary cutoff thresholds. By contrast, our study provides real-world evidence from a Chinese single-center CIN2/3 cohort, evaluating excision depth as a continuous variable across multiple sensitivity analyses.

The findings for margin status are broadly consistent with studies indicating that increasing excision depth does not necessarily translate into better treatment-related outcomes. Lara-Peñaranda et al. reported that LLETZ excisions of 10 mm or less were not associated with less favorable disease or HPV outcomes, while a more recent cohort found margin clearance to be more informative than excision depth for predicting test-of-cure outcomes (12, 13). Our findings extend these observations by evaluating excision depth as a continuous measure and by examining the association across several clinically plausible definitions of margin positivity and different depth specifications. At the same time, previous studies have reported a greater likelihood of negative margins with increasing excision length, particularly in women with type 3 transformation zones (9–11). These findings are not necessarily inconsistent. The depth required to achieve complete excision is likely to vary according to transformation-zone anatomy, lesion extent, endocervical involvement, and procedural technique. In the present single-center cohort, the median LEEP excision depth was relatively shallow (median 6 mm; IQR 5–8 mm), with few patients undergoing very deep cervical resection. Accordingly, our null findings regarding excision-depth associations apply strictly to routine standard-practice LEEP cases within this relatively narrow depth range. These results cannot be extrapolated to clinical scenarios requiring deep endocervical resection, such as lesions associated with Type 3 transformation zones, for which deeper conization remains clinically necessary and indicated.

Recorded postoperative hrHPV positivity was observed in 22.2% of the secondary analytical cohort. This proportion is broadly comparable with the median 6-month HPV persistence reported in a previous systematic review, although the present outcome should not be considered equivalent to 6-month persistence because the interval between LEEP and postoperative HPV testing could not be uniformly established (8). Age emerged as the most consistent clinical correlate of recorded postoperative hrHPV positivity in our analyses. This finding is in line with previous follow-up studies showing that increasing age may contribute to heterogeneity in post-treatment HPV persistence and subsequent disease risk, potentially driven by age-related transformation-zone retraction and immunosenescence (15, 16). By contrast, preoperative HPV16/18 status was not independently associated with recorded postoperative hrHPV positivity in the current cohort, whereas a recent meta**-**analysis reported a higher likelihood of persistent infection among women with HPV16 (17). Differences in genotype classification, timing of postoperative testing, and patterns of follow-up may partly account for these differences.

Margin status also showed a relatively imprecise association with recorded postoperative hrHPV positivity in patients for whom both outcomes were available. This finding should be interpreted in the context of the limited number of positive margins in the joint analytical subset and does not diminish the established clinical importance of margin status. Previous pooled evidence has consistently linked positive margins with persistent HPV infection and treatment failure (5, 6, 17). Of note, the exploratory pathway analysis in our study showed that the standardized difference in recorded postoperative hrHPV probability between 8-mm and 5-mm excision depths was largely retained after accounting for margin status, whereas the margin-associated indirect component was close to zero. Within the constraints of the available data, this pattern suggests that any relationship between excision depth and recorded postoperative hrHPV status is unlikely to be explained solely by recorded margin status. However, because this analysis was exploratory and the timing of postoperative HPV testing was not standardized, it should be regarded as hypothesis-generating rather than evidence of a causal pathway.

The clinical implications of these findings relate to the balance between adequate excision and preservation of cervical tissue. More extensive cervical excision may reduce the risk of incomplete treatment in selected patients but is also associated with an increased risk of subsequent preterm birth (4). When residual disease is suspected, subsequent secondary excisional treatment may be required, which itself carries elevated risks for adverse obstetric outcomes. Our study was not designed to test or validate individualized excision strategies, nor did we evaluate whether such approaches improve oncological or reproductive outcomes. Taken together, and within the bounds of our observational data, our findings do not support uniformly maximizing excision depth as a strategy. We interpret them as supporting consideration of excision depth alongside lesion characteristics, transformation-zone anatomy, reproductive considerations, and procedural judgement, rather than as a recommendation to alter current surgical practice. Current guidelines and pooled evidence support post**-**treatment HPV testing as a central component of risk assessment after excisional treatment (2, 3, 6, 7). In this context, excision depth may contribute useful procedural information, but it should not substitute for margin interpretation or guideline-based postoperative surveillance.

Several aspects of the present study strengthen the interpretation of the findings. Excision depth was evaluated continuously rather than being reduced to an arbitrary binary threshold, and we examined whether the main estimates were sensitive to a range of reasonable analytical choices. These choices included alternative margin definitions, nonlinear modeling, specification analyses, outcome-observation weighting, and prespecified interaction analyses. The depth estimates were consistent across these approaches, although all analyses share the limitations inherent to this retrospective dataset.

Several limitations should nevertheless be considered. First, the retrospective design relied on clinical and pathological information recorded across existing institutional data sources, and the availability of individual variables was not uniform.

Substantial sample attrition occurred from the initial pool of 1, 363 consolidated patient candidates. A baseline comparison between included and excluded participants is provided in **Supplementary Table S2**. Excluded patients had a lower proportion of CIN3 (9.5% vs 28.7%; P < 0.001) and, among those with available data, a lower proportion of high-grade cytology (25.9% vs 32.5%; P = 0.046), whereas age, excision depth, and preoperative HPV16/18 status were similar between groups (P = 0.389, 0.584, and 0.407, respectively). Because IPOW can adjust only for missingness related to measured baseline covariates, residual selection bias related to unmeasured determinants of clinical follow-up and documentation practice, as well as confounding by indication, cannot be completely ruled out given the retrospective design.

A dedicated baseline comparison between patients with versus without available postoperative hrHPV test results is provided in **Supplementary Table S3**. Baseline characteristics were largely balanced between the two groups despite a modest imbalance in excision depth, which was accounted for within the IPOW sensitivity analyses.

Second, postoperative hrHPV results were unavailable for a substantial proportion of the core cohort. In summary, because postoperative testing windows varied widely and preoperative-to-postoperative genotype tracking was unavailable, recorded postoperative hrHPV positivity serves strictly as an exploratory marker of viral detection at a nonstandardized time point rather than as verified persistent infection, treatment failure, or cure.

Third, several potentially relevant anatomical and clinical characteristics were not available, including transformation-zone type, lesion dimensions, parity, smoking status, immune status, loop size, operator characteristics, specimen fragmentation, and details of pathological processing. We were unable to stratify margin outcomes by anatomical margin location (endocervical versus ectocervical versus deep margins), because standardized separate reporting for distinct margin sites was not consistently available across pathology records. The absence of these variables limits our ability to determine whether the effect of excision depth differs across specific anatomical or procedural settings. These surgical-related variables could not be incorporated into our regression adjustment.

Fourth, pathology-recorded specimen depth may not correspond exactly to the intended *in vivo* excision depth because of tissue handling, specimen shrinkage, and procedural estimation. Previous work has demonstrated clinically relevant discrepancies between presumed and measured excision depth (18), and such measurement variability may reduce the apparent strength of depth–outcome associations. Our cohort contains relatively few patients with very deep excision (>12 mm); therefore our findings cannot exclude associations in cohorts with a larger proportion of extensive resection for type 3 transformation-zone lesions.

Finally, the number of events was smaller in the extended models, and these analyses should therefore be interpreted primarily as supportive sensitivity analyses. The exploratory pathway analysis was based on a more restricted subset and was not designed to establish causal mediation. In addition, reliable recurrence dates and censoring information were not available, preventing direct evaluation of residual or recurrent CIN2+ and time-to-event treatment failure. Future prospective studies incorporating standardized HPV testing intervals, detailed transformation-zone characteristics, lesion dimensions, and longer-term recurrence outcomes would help clarify whether excision depth has greater clinical relevance within specific patient or anatomical subgroups.

In conclusion, in our study population with a relatively narrow range of pathology**-**recorded excision depths, pathology**-**recorded LEEP excision depth provided limited independent statistical information regarding margin positivity and recorded postoperative hrHPV positivity in women with CIN2/3, whereas histopathologic grade and age showed more consistent associations with these respective outcomes. Within the constraints of this single-center retrospective cohort, these findings do not support the use of excision depth alone as a surrogate for treatment adequacy, and we interpret them as supporting a broader clinical assessment that integrates lesion characteristics, pathological findings, reproductive considerations, and postoperative HPV surveillance.

## Data Availability

The data analyzed in this study is subject to the following licenses/restrictions: The deidentified participant data that support the findings of this study are not publicly available because they were obtained from institutional clinical and pathology records under the ethics approval granted for this study, and the terms of that approval restrict public sharing of the underlying patient-level data. The deidentified analysis-ready dataset and the statistical code used to generate the results are available from the corresponding author upon reasonable request, subject to approval by the Hainan General Hospital Ethics Committee and execution of the required data-use agreement. Requests to access these datasets should be directed to GL, 15203658656@139.com.

## Supplementary material

Supplementary Table 1 Sensitivity analysis for preoperative HPV reference-group composition Note: Core multivariable logistic regression, adjusted for age, histopathologic grade, and preoperative HPV16/18 status. Only 7 of the 422 patients in the primary margin cohort (1.7%) were preoperatively HPV-negative, so a separate HPV-negative stratum was not estimable; heterogeneity arising from pooling non-16/18 hrHPV-positive and HPV-negative patients was therefore evaluated by excluding the latter from the reference group. None of these 7 patients had a positive margin, so their exclusion left the depth-related estimates essentially unchanged. In the other-hrHPV-positive subgroup, preoperative HPV16/18 status was constant across all patients and was therefore not included as a covariate. hrHPV, high-risk human papillomavirus; OR, odds ratio.

Supplementary Table 2 Baseline comparison of included versus excluded participants. Abbreviations: LEEP, loop electrosurgical excision procedure; CIN, cervical intraepithelial neoplasia; HPV, human papillomavirus; IQR, interquartile range. Note: Data are median (IQR) or n (%). Percentages for excision depth, preoperative HPV16/18 status, and high-grade cytology use the available denominator within each group (available denominators among the 875 excluded patients: excision depth 261, preoperative HPV16/18 status 596, high-grade cytology 424); age was available for 488 and 874 patients, respectively, and cytology data were available for 335 of the 488 included patients. For the CIN3 comparison, the full group was used as the denominator: the 236 excluded patients with unresolved (n = 234) or conflicting (n = 2) pathology grades were classified as non-CIN3 for this descriptive comparison. Gland involvement was recorded for 482 of the 488 included patients but for only 290 of the 875 excluded patients; because of this limited and selectively missing documentation, gland involvement was not compared between groups. P values were calculated with the Mann–Whitney U test for continuous variables and the Pearson χ² test for categorical variables; no cell had an expected frequency below 5, so the Fisher exact test was not required.

Supplementary Table 3 Baseline comparison of core-cohort patients with versus without recorded postoperative hrHPV data Abbreviations: hrHPV, high-risk human papillomavirus; LEEP, loop electrosurgical excision procedure; CIN, cervical intraepithelial neoplasia; IQR, interquartile range. Note: Data are median (IQR) or n (%), unless otherwise indicated. ^a^Percentage based on gland-involvement data available for 271 and 211 patients in the two groups, respectively. ^b^Percentage based on high-grade cytology data available for 197 and 138 patients in the two groups, respectively. P values were calculated with the Mann–Whitney U test for continuous variables and the Pearson χ² test for categorical variables; no cell had an expected frequency below 5, so the Fisher exact test was not required. The CIN grade P value refers to the overall 2×2 comparison of CIN2 versus CIN3 between the two groups. The single statistically significant difference was LEEP excision depth, which was modestly greater among patients with recorded postoperative hrHPV data (P = 0.001), a pattern consistent with more intensive documentation or clinical follow-up among patients undergoing more extensive procedures; this measured imbalance was explicitly incorporated into the inverse-probability-of-observation-weighted (IPOW) sensitivity analyses.
