# Supplementary material for: LEEP excision depth is not independently associated with margin positivity or recorded postoperative hrHPV positivity in women with CIN2/3: a retrospective cohort study
Source: Front Oncol. 2026 Oct 27;16:1969282. doi: 10.3389/fonc.2026.1969282 (PMC13748206; doi:10.3389/fonc.2026.1969282)
Supplement: Supplementary file 1 [file DataSheet1.pdf]

Supplementary Material

LEEP Excision Depth Is Not Independently Associated With Margin Positivity or Recorded Postoperative hrHPV Positivity in Women With CIN2/3: A Retrospective Cohort Study

Jie Cheng, Chunyan Hu, Genhai Zhu, Fujin Liu, Guifei Li

Supplementary Table S1. Sensitivity analysis for preoperative HPV reference-group composition

Model subgroup	N	Adjusted OR for excision depth (per 3-mm increase)	95% CI	P value
Primary margin cohort, full model (reference group: pooled non-16/18 hrHPV-positive and HPV-negative patients)	422	0.95	0.72–1.26	0.74
Primary margin cohort, preoperatively HPV-negative patients excluded	415	0.95	0.72–1.26	0.74
Subgroup: other-hrHPV-positive patients only	214	0.87	0.52–1.44	0.57

Data are from core multivariable logistic regression models adjusted for age, histopathologic grade, and preoperative HPV16/18 status. Only 7 of the 422 patients in the primary margin cohort (1.7%) were preoperatively HPV-negative, so a separate HPV-negative stratum was not estimable; heterogeneity arising from pooling non-16/18 hrHPV-positive and HPV-negative patients was therefore evaluated by excluding the latter from the reference group. None of these 7 patients had a positive margin, so their exclusion left the depth-related estimates essentially unchanged. In the other-hrHPV-positive subgroup, preoperative HPV16/18 status was constant across all patients and was therefore not included as a covariate. **Abbreviations:** hrHPV, high-risk human papillomavirus; OR, odds ratio.

Supplementary Table S2. Baseline comparison of included versus excluded participants

Characteristic	In core-covariate cohort (N = 488)	Excluded from core-covariate cohort (N = 875)	P value
Age, years, median (IQR)	39.0 (34.0–48.0)	39.0 (32.0–48.0)	0.389
LEEP excision depth, mm, median (IQR)	6.0 (5.0–8.0)	6.0 (5.0–8.0)	0.584
CIN3, n (%)	140 (28.7)	83 (9.5)	<0.001

Characteristic	In core-covariate cohort (N = 488)	Excluded from core-covariate cohort (N = 875)	P value
Preoperative HPV16/18 positive, n (%)	208 (42.6)	269 (45.1)	0.407
High-grade cytology, n (%)	109 (32.5)	110 (25.9)	0.046

Data are median (IQR) or n (%), unless otherwise indicated. Percentages for excision depth, preoperative HPV16/18 status, and high-grade cytology use the available denominator within each group (available denominators among the 875 excluded patients: excision depth 261, preoperative HPV16/18 status 596, high-grade cytology 424); age was available for 488 and 874 patients, respectively, and cytology data were available for 335 of the 488 included patients. For the CIN3 comparison, the full group was used as the denominator: the 236 excluded patients with unresolved (n = 234) or conflicting (n = 2) pathology grades were classified as non-CIN3 for this descriptive comparison. Gland involvement was recorded for 482 of the 488 included patients but for only 290 of the 875 excluded patients; because of this limited and selectively missing documentation, gland involvement was not compared between groups. P values were calculated with the Mann–Whitney U test for continuous variables and the Pearson χ^2 test for categorical variables; no cell had an expected frequency below 5, so the Fisher exact test was not required. **Abbreviations:** LEEP, loop electrosurgical excision procedure; CIN, cervical intraepithelial neoplasia; HPV, human papillomavirus; IQR, interquartile range.

Supplementary Table S3. Baseline comparison of core-cohort patients with versus without recorded postoperative hrHPV data

Characteristic	Recorded postoperative hrHPV data (n = 275)	No recorded postoperative hrHPV data (n = 213)	P value
Age, years, median (IQR)	39.0 (34.0–47.0)	39.0 (33.0–50.0)	0.948
LEEP excision depth, mm, median (IQR)	7.0 (5.0–8.0)	6.0 (5.0–8.0)	0.001
CIN grade, n (%)			0.823
CIN2	195 (70.9)	153 (71.8)	
CIN3	80 (29.1)	60 (28.2)	
Preoperative HPV16/18 positive, n (%)	111 (40.4)	97 (45.5)	0.251

Characteristic	Recorded postoperative hrHPV data (n = 275)	No recorded postoperative hrHPV data (n = 213)	P value
Gland involvement, n (%)	163 (60.1)[a]	138 (65.4)[a]	0.237
High-grade cytology, n (%)	61 (31.0)[b]	48 (34.8)[b]	0.463

Data are median (IQR) or n (%), unless otherwise indicated. [a]Percentage based on gland involvement data available for 271 and 211 patients in the two groups, respectively. [b]Percentage based on high-grade cytology data available for 197 and 138 patients in the two groups, respectively. P values were calculated with the Mann–Whitney U test for continuous variables and the Pearson χ^2 test for categorical variables; no cell had an expected frequency below 5, so the Fisher exact test was not required. The CIN grade P value refers to the overall 2×2 comparison of CIN2 versus CIN3 between the two groups. The single statistically significant difference was LEEP excision depth, which was modestly greater among patients with recorded postoperative hrHPV data (P = 0.001), a pattern consistent with more intensive documentation or clinical follow-up among patients undergoing more extensive procedures; this measured imbalance was explicitly incorporated into the inverse-probability-of-observation-weighted (IPOW) sensitivity analyses. **Abbreviations:** hrHPV, high-risk human papillomavirus; LEEP, loop electrosurgical excision procedure; CIN, cervical intraepithelial neoplasia; IQR, interquartile range.
